# Integrated Puncture Score: force–displacement weighted rind penetration tests improve stalk lodging resistance estimations in maize

**DOI:** 10.1186/s13007-020-00654-w

**Published:** 2020-08-15

**Authors:** Christopher J. Stubbs, Christopher McMahan, Will Seegmiller, Douglas D. Cook, Daniel J. Robertson

**Affiliations:** 1 Department of Mechanical Engineering, University of Idaho, Moscow, ID 83844 USA; 2 School of Mathematical and Statistical Sciences, Clemson University, Clemson, SC 29634 USA; 3 Department of Mechanical Engineering, Brigham Young University, Provo, UT 84602 USA

**Keywords:** Integrated, Lodging, Maize, Phenotyping, Plant, Rind, Puncture, Penetration, Stalk, Stem, Strength

## Abstract

**Background:**

Stalk lodging (breaking of agricultural plant stalks prior to harvest) is a multi-billion dollar a year problem. Rind penetration resistance tests have been used by plant scientists and breeders to estimate the stalk lodging resistance of maize for nearly a hundred years. However, the rind puncture method has two key limitations: (1) the predictive power of the test decreases significantly when measuring elite or pre-commercial hybrids, and (2) using rind penetration measurements as a breeding metric does not necessarily create stronger stalks. In this study, we present a new rind penetration method called the Integrated Puncture Score, which uses a modified rind penetration testing protocol and a physics-based model to provide a robust measure of stalk lodging resistance.

**Results:**

Two datasets, one with a diverse array of maize hybrids and one with only elite hybrids, were evaluated by comparing traditional rind penetration testing and the Integrated Puncture Score method to measurements of stalk bending strength. When evaluating the diverse set of hybrids, both methods were good predictors of stalk bending strength (R^2^ values of 0.67). However, when evaluating elite hybrids, the Integrated Puncture Score had an R^2^ value of 0.74 whereas the traditional method had an R^2^ value of 0.48. Additionally, the Integrated Puncture Score was able to differentiate between the strongest and weakest hybrids in the elite hybrid data set whereas the traditional rind penetration method was not. Additional experiments revealed strong evidence in favor of the data aggregation steps utilized to compute the Integrated Puncture Score.

**Conclusions:**

This study presents a new method for evaluating rind penetration resistance that highly correlates with stalk bending strength and can possibly be used as a breeding index for assessing stalk lodging resistance. This research lays the foundation required to develop a field-based high-throughput phenotyping device for stalk lodging resistance.

## Background

Stalk lodging (permanent displacement of plants from their vertical orientation) severely reduces agronomic yields of several vital crop species including maize [1–6].Yield losses due to stalk lodging are estimated to range from 5–20% annually [7, 8]. Stalk lodging, as opposed to root lodging, occurs when the mechanical stability of the plant is lost due to structural failure of the plant stem [5, 10–13].

To estimate stalk strength and stalk lodging resistance of large grain crops plant scientist frequently utilize rind puncture tests [14–29]. Despite nearly 100 years of research the rind puncture method remains virtually unchanged from the time at which it was first introduced to the research community. In particular, the method consists of simply measuring the peak penetration force required to insert a probe through a plant's rind. The underlying assumption is that the penetration force is related to the material properties of the rind tissue which is in turn related to stalk bending strength / lodging resistance. Numerous researchers have demonstrated that rind puncture resistance measurements correlate with stalk lodging resistance [15, 17, 18, 29, 30].

However, the rind puncture method has not been widely adopted by breeding programs and it suffers from 2 key limitations. First, although rind penetration measurements have been shown to correlate with stalk lodging, the predictive power of the test decreases significantly when measuring elite or pre-commercial hybrids thus limiting its utility in late stage breeding trials [29, 31, 32]. Second, using rind penetration measurements as a breeding metric does not necessarily create stronger stalks [15, 29]. For example, repeated selection for rind penetration resistance has been shown to produce stalks with smaller diameters [15]. Stalks with smaller diameters are known to be structurally inferior to stalks with larger diameters [32–34]. Thus, using rind penetration resistance as a selective breeding metric can produce stalks with a structurally disadvantageous morphology. In other words, rind penetration measurements do not measure or account for cross-sectional geometries or the spatial distribution of material stiffness within the plant, both of which are known to be highly correlated with stalk lodging resistance [32, 35].

The purpose of this study is to present a methodology for a modified rind penetration measurement that addresses these limitations by integrating both the tissue stiffness and the distribution of that stiffness into a single measurement called the ‘Integrated Puncture Score’. It is anticipated that the new method will enable plant breeders to use rind penetration tests to (1) better assess elite hybrids for stalk lodging resistance and (2) be used directly as a selective breeding index to improve stalk lodging resistance.

## Methods

### Experimental materials

Two unique sets of maize hybrids were utilized in this study. The first set of hybrids were selected to represent a reasonable portion of maize genetic diversity and morphology. The second set consisted solely of elite commercial hybrids. The first set was chosen to mimic the type of diversity encountered when conducting diversity panel experiments. The second set was chosen to mimic the type of diversity encountered in late stage pre-commercial breeding trials. Hereafter the first set will be referred to as the “Diversity Set” and the second set will be referred to as the “Commercial Set”. More information about each set of hybrids and the sampling strategy for each set is given below.

The Diversity Set of maize stalks was chosen to represent a reasonable portion of maize genetic diversity and were selected for variation in stem morphology and biomass distribution. The hybrids were planted at Clemson University Simpson Research and Education Center, Pendleton, SC in well drained Cecil sandy loam soil. The hybrids were grown in a Random Complete Block Design with two replications. In each replication, each hybrid was planted in two-row plots with row length of 4.57 m and row-to-row distance of 0.76 m with a targeted planting density of 70,000 plant ha^−1^. The experiment was surrounded by non-experimental maize hybrids on all four sides to prevent any edge effects. To supplement nutrients, 56.7 kg ha^−1^ nitrogen, 86.2 kg ha^−1^ of phosphorus and 108.9 kg ha^−1^ potassium was added at the time of soil preparation, and an additional 85 kg ha^−1^ nitrogen was applied 30 days after emergence. Standard agronomic practices were followed for crop management.

The Commercial Set of maize stalks consisted of five commercial varieties of dent corn grown during the 2013 season at Monsanto facilities in Iowa in a randomized block design which included planting densities of 119,000, 104,000, 89,000, 74,000, and 59,000 plants ha–1 (48,000, 42,000, 36,000, 30,000, and 24,000 plants ac–1), 2 locations, and two replicates. Additional information about the origin and sampling of these stalks can be found in a previous report [32].

All stalks used for this study were harvested when all the hybrids were either at or past physiological maturity (i.e., 40 days after anthesis). Ten competitive plants from each plot were harvested by cutting them just above ground level, removing all the leaves and ears, and finally transferring them to a forced air dryer for drying. Stalks were dried to mitigate the confounding effects of moisture content and turgor pressure. In addition, the authors were primarily interested in the problem of late season stalk lodging which occurs when stalks are fully mature and dry. Drying stalks prior to testing is in line with other studies performed on late season lodging [14, 29, 32, 34–38]. Another key advantage of using dried stalks is that their material properties do not change over time thus enabling storage of stalk samples. Some plots lacked 10 competitive plants and, therefore, the total number of plants evaluated for each hybrid varied slightly. In total, 841 (Diversity Set) and 933 (Commercial Set) fully mature, dried maize stalks were used in this study. All stalks included in the study (from both the Diversity and Commercial Sets) were submitted to three-point bending and rind penetration tests as described below.

### Three-point bending

Three-point bending tests were performed on all stalk specimens. A Universal Testing System (Instron Model # 5944, Norwood MA) was used to perform the tests. Stalks were loaded at nodes to avoid premature local failure because of cross sectional compression in the weaker internodal regions [9, 39]. Each stalk was supported on their uppermost and lowermost (apical to basal) nodes. Specimens were loaded until failure, and the maximum bending moment was recorded. Load–displacement data was collected using Bluehill Universal Testing Software (Illinois TookWorks Inc., Glenview IL). Further details on the three-point bending method can be found in [9, 40].

### Rind puncture testing

**Fig. 1:**
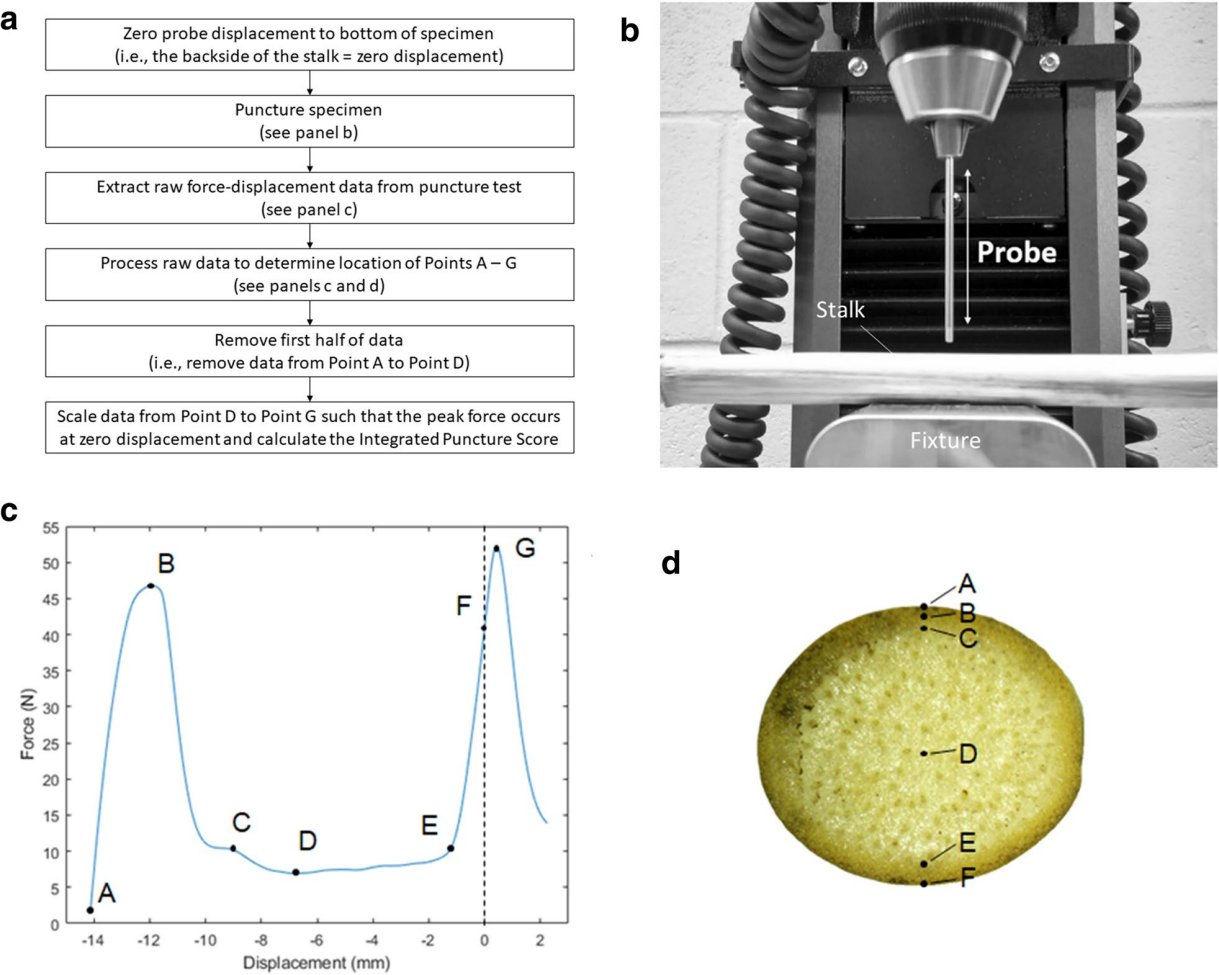
**a** Flow chart of the process used to calculate the Integrated Puncture Score. **b** Image of the rind puncture test setup. The fixture supporting the plant sample has a 10 mm diameter hole, in it to allow the probe to puncture through the entire stalk without hitting the support fixture. The images were adapted from [29]. **c** A typical load–displacement curve resulting from a rind penetration test; key points on the plot are as follows: Point A—probe entry point, Point B—first force peak, Point C—first rind-pith transition region, Point D – midpoint of stalk cross-section, Point E—second rind-pith transition region, Point F—precalibrated zero displacement plane (i.e., the bottom of the test specimen / top of the support fixture), Point G—second force peak. **d** A cross-section of a test specimen with the key points labeled

Rind puncture tests were performed on all stalk specimens. In particular, a Universal Testing System (Instron, model # 5944, Norwood MA) was used to puncture the centermost internode of each stalk sample in the direction of the minor cross-sectional axis (i.e., in the direction of the minor diameter of the stalk) with a stainless steel probe. The probe was 2 mm in diameter with a 45 degree 0.5 mm chamfer on its end. The probe was lowered until it had completely punctured the entirety of the stalk cross-section. Note this is slightly different than a typical rind puncture test. In a traditional rind penetration test the probe is typically retracted after reaching the center of the stalk cross-section and the maximum force is recorded. In this study synchronous load and displacement data from each penetration test were acquired using Bluehill Universal Testing Software (Illinois TookWorks Inc., Glenview IL). Load–displacement data were acquired at a rate of 1000 samples per second and the probe was actuated at a rate of 25 mm/s. An image of the test setup is shown in Fig. 1a. Further details on the puncture method and probe geometry can be found in previous studies from our lab [14, 29]. It should be noted that while rind penetration testing is quite common there are no commonly agreed methods or protocols for conducting rind penetration tests in the literature [29]. Thus, different studies frequently use different penetration instruments, puncture rates and probe geometries. For this study the ‘traditional rind puncture measurement’ was attained by determining the maximum load (i.e. force) that occurred in the puncture test prior to the tip of the probe passing the midpoint of the stalk cross-section. The puncture rate, probe geometry and test setup were chosen based on recommendations presented in [29]. The Integrated Puncture Score was calculated as described below.

### Integrated Puncture Score

The Integrated Puncture Score for each stalk was calculated using a custom Matlab algorithm. The algorithm was developed using structural engineering principles and theory that govern the flexural response of engineering structures. In particular the algorithm was designed to simultaneously account for the cross-sectional distribution and puncture strength of stalk tissues. The underlying theory and mechanics of the algorithm is described below. The source code for the algorithm has been uploaded as Additional file 1. Additional files 2-6 contain example rind puncture test data that can be used in conjuction with the Integrated Puncture Score algorithim.

Figure 1a displays a flowchart which outlines the process used to calculate the Integrated Puncture Score. Figure 1b shows an image of the experimental test setup. A typical load–displacement curve from a rind puncture test of a maize stalk is shown in Fig. 1c. As shown in Fig. 1 the penetrating probe makes initial contact with the stalk specimen at (Fig. 1c—Point A). After initial contact the load rapidly increases until the probe penetrates the rind tissue (Fig. 1c—Point B). A rapid decrease in load is observed as the probe begins to enter the pith tissues (Fig. 1c—Point C). The load maintains a relatively low force as the probe is driven through the specimen’s pith (Fig. 1c—Points C to E). When the probe engages with the rind tissues on the far side of the stalk cross-section the load rapidly increases again (Fig. 1c—Point E). The tip of the probe typically passes the pre-calibrated zero-deflection point (i.e., the back side of the stalk cross-section, Fig. 1c—Point F), and continues increasing in load until it breaks through the far-side of the specimen (Fig. 1c—Point G). Note the peak force does not necessarily coincide with the Point F. This is due to complex fracture mechanics, rapid crack propagation, and slight deflections of the rind tissue that occur during puncture testing. The Integrated Puncture Score algorithm extracts these points using peak identification and slope thresholding algorithms as described in a previous study from our lab [29] Additional file 2.

Once these points have been identified, the Integrated Puncture Score algorithm performs several additional pre-analysis steps. First, the midpoint of the stalk cross-section (Fig. 1—point D) is defined as lying halfway between Points A and F. Data from the initial contact of the probe with the stalk (Fig. 1—Point A) to the midpoint of the stalk cross-section (Fig. 1—Point D) is then removed. Second, the data from the midpoint (Fig. 1—Point D) to the peak load (Fig. 1—Point G) is scaled in the x-direction such that Point G (the peak load) will coincide with the zero-plane (Fig. 1—Point F). The data was transformed because the Integrated Puncture Score heavily weights data near the zero plane (e.g., the puncture force is weighted by distance from midpoint to the fourth power). Thus, small inconsistencies between samples near the zero plane (e.g., different locations of max force) get amplified as they are raised to the fourth power. Therefore, the authors decided to transform the data as described above to provide a more ‘normalized’ / more comparable force–displacement curve for each sample. Figure 1d displays a typical stalk cross-section with labeled points corresponding to points A–F in Fig. 1c, Additional file 3.

To calculate the Integrated Puncture Score, the scaled data (Fig. 1c—Point D to Point G) are numerically integrated to derive a material weighted section modulus analog. A typical material-weighted section (S_E_) modulus calculation of a heterogenous material would take the form [39] Additional file 4:

$$s_{E} = \frac{{\int\limits_{A} E x^{2} dA}}{{x_{\max } }}$$

where E is the tissue stiffness, and x is the distance of that tissue to the neutral bending layer of the structure in question with *x* having a maximum value denoted as x_max_. A similar approach is used to calculate the Integrated Puncture Score. In particular, we calculate the Integrated Puncture Score by numerically integrating the transformed load–displacement curve from the puncture test using the penetrating force as an approximate measure of tissue stiffness or strength. In other words, the penetrating force is weighted by the fourth power of the distance to the neutral layer (Fig. 1c—point D) Additional file 5:

$$IPS = {{\left( {\sum\limits_{{n = Po{\text{int}} \,D}}^{{Po{\text{int}} \,G}} {F_{n} \cdot x_{n}^{4} - F_{n - 1} \cdot x_{n - 1}^{4} } } \right)} \mathord{\left/ {\vphantom {{\left( {\sum\limits_{{n = Po{\text{int}} \,D}}^{{Po{\text{int}} \,G}} {F_{n} \cdot x_{n}^{4} - F_{n - 1} \cdot x_{n - 1}^{4} } } \right)} {x_{\max } }}} \right. \kern-\nulldelimiterspace} {x_{\max } }}$$

where the resulting value matches Eq. 1 in units of *puncture force* x *length*^3^.

It is worth noting the primary difference between the Integrated Puncture Score and traditional rind penetration methods lies in the processing of the data. Traditional rind penetration is calculated as the maximum force on the initial penetrating event (i.e. the force value at Fig. 1—Point B). In other words, no displacement data are collected or utilized during a typical rind penetration test. In contrast the Integrated Puncture Score is calculated by numerical integrating the transformed load–displacement data from Fig. 1—Point D to Point G, Additional file 6.

### Empirical model

To confirm the Integrated Puncture Score is an efficient and appropriate aggregation of the observed force curve data, we analyze the same using a functional regression model. The premise, the proposed functional regression model holds the form of the Integrated Puncture Score as a special case. Thus, if the fitted value of the functional regression model coincides with the Integrated Puncture Score then this validates it as the best aggregation of the observed information. To this end, let *Y*_*i*_ denote the strength measurement taken on the *i*th stalk, for *i* = 1*, …, m*. Further, let *F*_*i*_(*x*) denote the corresponding force curve at the *x*th position. To relate strength to the force curve we posit the following functional regression model.

3 $$Y_{i} = \gamma_{0} + \smallint \beta \left( x \right)F_{i} \left( x \right)dx +_{i} ,$$

where ${\epsilon }_{i}$, for $i = 1, \ldots ,m$, are homoscedastic mean-zero random errors that are uncorrelated with each other, ${\gamma }_{0}$ is an intercept parameter, and $\beta \left(x\right)$ is an unknown functional coefficient; for further discussion on functional regression models see Ramsay and Silverman (2007). It is important to note that $\beta \left(x\right)$ is an infinite dimensional parameter. Thus, to reduce the dimensionality of the problem, we approximate this parameter via B-splines (Schumaker, 2007); i.e., as.

4 $$\beta \left( x \right) = \mathop \sum \limits_{j = 1}^{J} B_{j} \left( x \right)\gamma_{j}$$

where ${B}_{j}\left(x\right)$ is a B-spline basis function and ${\gamma }_{j}$ is the corresponding spline coefficient, for *j* = 1*, …, J*. These basis functions are fully determined once a knot sequence and degree are specified; for further discussion see Schumaker (2007). For adequate modeling flexibility, in this application we use a knot set consisting of 7 interior knots (placed at equally spaced quantiles) and specified the degree to be 3. To smoothly estimate the functional coefficient, we use a regularizing penalty; i.e., our objective function takes on the form.

5 $$\hat{\user2{\gamma }}_{\lambda } = argmin_{{\varvec{\gamma}}} \mathop \sum \limits_{i = 1}^{m} \left\{ {Y_{i} - \gamma_{0} + \smallint \beta \left( x \right)F_{i} \left( x \right)dx} \right\}^{2} + \lambda \smallint \left\{ {\beta^{\left( 1 \right)} \left( x \right)} \right\}^{2} dx$$

where ${\varvec{\gamma}}=({\gamma }_{0},{\gamma }_{1},\dots ,{\gamma }_{J})\boldsymbol{^{\prime}}$ is the collection of unknown parameters, $\lambda$ is a penalty parameter, ${\widehat{{\varvec{\gamma}}}}_{\lambda }$ is a penalty parameter specific estimator of ${\varvec{\gamma}}$, and ${\beta }^{(1)}\left(x\right)$ is the first derivative of $\beta \left(x\right)$. To choose the penalty parameter we first note that.

6 $$\hat{\user2{\gamma }}_{\lambda } = \left\{ {\user2{M^{\prime}M} + {\varvec{R}}^{*} \left( \lambda \right)} \right\}^{ - 1} \user2{M^{\prime}Y}$$

where ${\varvec{Y}}=({Y}_{1},\dots ,{Y}_{m})\boldsymbol{^{\prime}}$, ${\varvec{M}}=({{\varvec{M}}}_{1}^{\boldsymbol{^{\prime}}},\dots ,{{\varvec{M}}}_{n}^{\boldsymbol{^{\prime}}})\boldsymbol{^{\prime}}$, ${{\varvec{M}}}_{i}=(1,{B}_{1}\left(x\right){X}_{i}\left(x\right),\dots ,{B}_{J}\left(x\right){X}_{i}\left(x\right)){^{\prime}}$, and ${{\varvec{R}}}^{*}(\lambda )$ is a $(J+1)\times (J+1)$ matrix whose first row and column are all zeros and whose remaining entries are given by ${{{\varvec{R}}}^{*}(\lambda )}_{j{j}^{^{\prime}}}=\lambda {B}_{j-1}^{(1)}\left(x\right){B}_{{j}^{^{\prime}}-1}^{(1)}\left(x\right)$. Thus, we chose the penalty parameter to be the value of $\lambda$ that minimizes the usual Schwartz Bayesian Information Criterion (BIC) with the “degrees of freedom” being specified as $df\left(\lambda \right)=tr({{\varvec{S}}}_{\lambda })$, where ${{\varvec{S}}}_{\lambda }= {\varvec{M}}{\{{{\varvec{M}}}^{^{\prime}}{\varvec{M}}+{{\varvec{R}}}^{*}(\lambda )\}}^{-1}{{\varvec{M}}}^{^{\prime}}$ and $tr({{\varvec{S}}}_{\lambda })$ denotes the trace of the matrix ${{\varvec{S}}}_{\lambda }.$

## Results

**Fig. 2:**
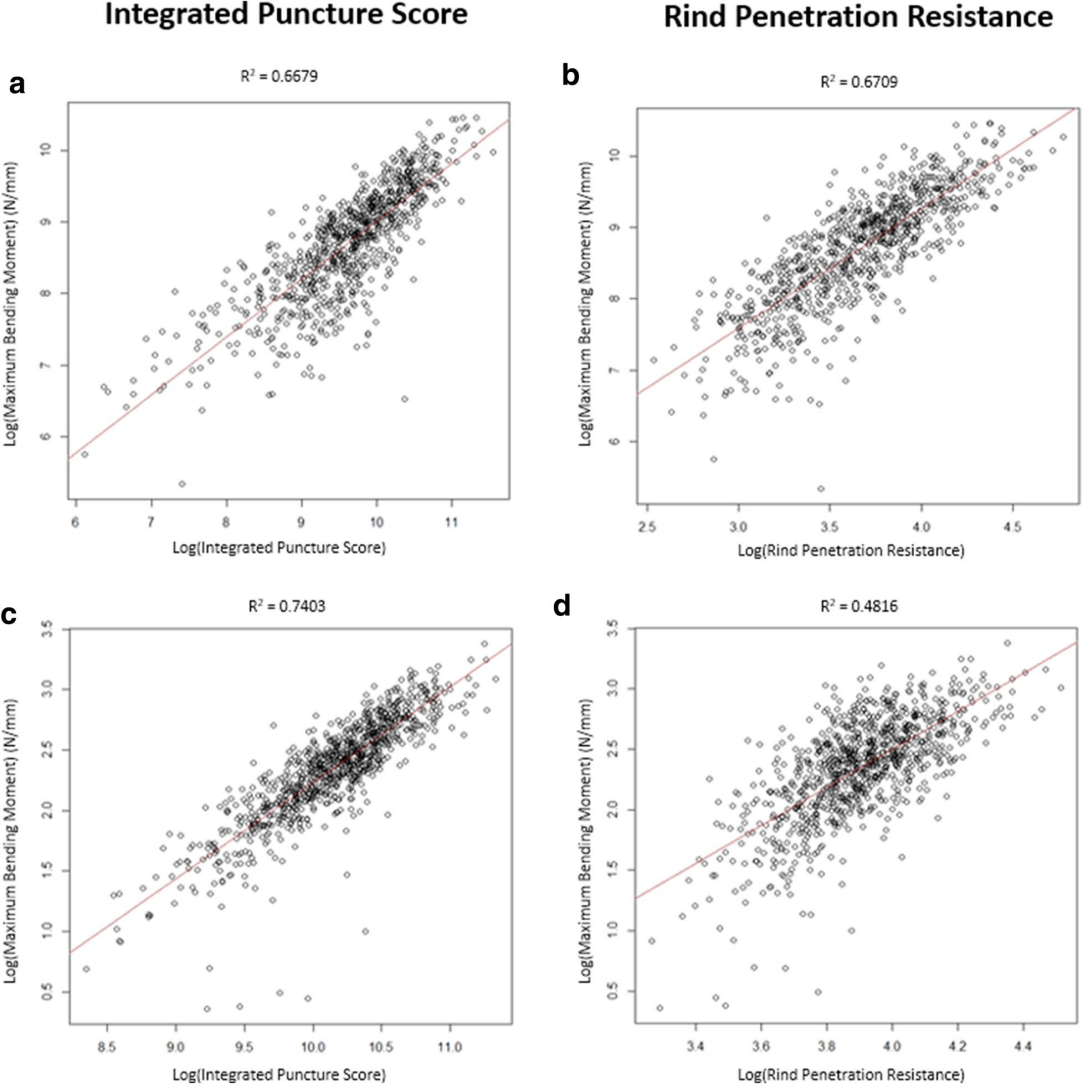
A linear regression model of log-bending strength with log-Integrated Puncture Score (**a**, **c**) and of log-bending strength with log-rind puncture resistance (**b**, **d**) for the Diversity Set of stalks (a, b) and the Commercial Set of stalks (**c**, **d**). Both the Integrated Puncture Score and the rind puncture resistance were good predictors of stalk bending strength for the Diversity Set of stalks. However, the Integrated Puncture Score was a much better predictor of stalk bending strength for the Commercial Set of stalks

To test the hypothesis that rind penetration tests predict stalk bending strength, a series of statistical analyses were performed. To formally examine this stated hypothesis, we posit and fit a linear regression model where log-rind-puncture-resistance or log-Integrated-Puncture-Score is the predictor variable and log-strength is the response variable of interest. Figure 2 depicts the results of these linear regressions. For the Diversity Set, we find that both Integrated Puncture Score (R^2^ = 0.67) and rind puncture resistance (R^2^ = 0.67) are associated with bending strength. For the Commercial Set, we find that as hypothesized the association with Integrated Puncture Score remains high (R^2^ = 0.74), but the association with traditional rind puncture resistance decreases (R^2^ = 0.48).

**Fig. 3:**
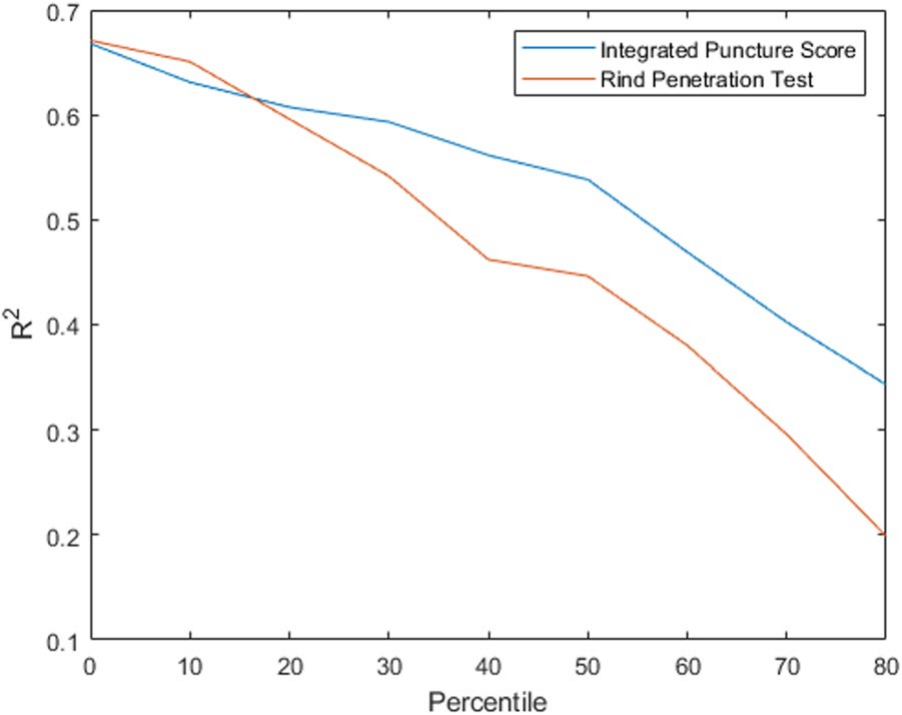
R^2^ values of the regression between log-rind puncture resistance with log-bending strength and log-Integrated Puncture Score with log-bending strength when removing the nth weakest percentile of stalks from the Diversity dataset (e.g. when “Percentile” is equal to 30, the linear regression is only performed on the strongest 70th percentile of stalks). Integrated Puncture Score has a stronger correlation than the traditional rind penetration tests and is more robust when looking at stronger, more elite plants

A further analysis was conducted to test the assertion that the Integrated Puncture Score is better able to distinguish elite hybrids for stalk lodging resistance than traditional rind puncture techniques. In particular, we reanalyzed the Diversity Set leaving out the nth weakest percentile, where n was allowed to range from 0–80 percent. In other words, the weakest stalks were systematically discarded from the analysis and the R^2^ values between stalk bending strength and each puncture test technique were reevaluated. Figure 3 depicts the R^2^ values of each technique as a function of n (percentile strength). As seen in Fig. 3 the Integrated Puncture Score demonstrates a stronger association with stalk bending strength especially when only elite specimens (i.e., strong stalks) are included in the analysis. This finding is discussed further in the Discussion section.

### Comparison of Integrated Puncture Score to empirical model

**Fig. 4:**
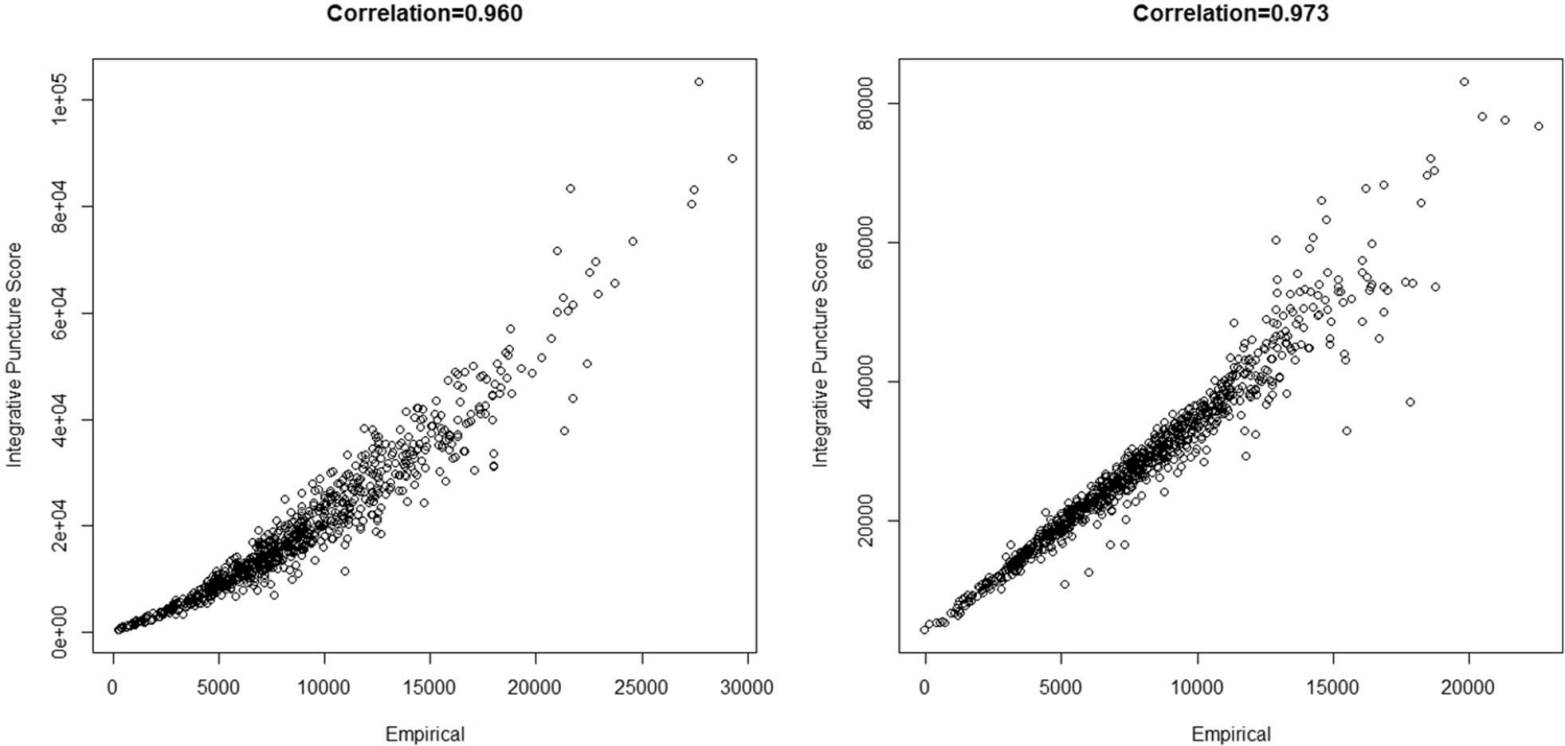
Scatter plot of the Integrated Puncture Score vs. fitted values arising from the empirical model for the Diversity Set (left) and Commercial Set (right) of maize stalks. The very tight correlation suggest that the Integrated Puncture Score effectively captures the features of the load–displacement curve from a puncture test that most closely relate to the bending strength of the test specimen

As a point of validation [41], we examine the hypothesis that the Integrated Puncture Score is the best way to aggregate the synchronous load–displacement data captured during a puncture test to explain stalk bending strength. This is evaluated by fitting the functional regression model (which holds the Integrated Puncture Score aggregation as a special case) to the strength data. The fitted values (i.e. the estimated value of the linear predictor from the functional regression analysis) is then compared to the Integrated Puncture Score. Figure 4 depicts the results of Integrated Puncture Score vs. the fitted values from the empirical functional regression analysis. It is found that the fitted values from the empirical model and the Integrated Puncture Score are highly correlated for both the Diversity Set (R^2^ = 0.92) and the Commercial Set (R^2^ = 0.94), which suggest two findings. First, the Integrated Puncture Score captures the features of the load–displacement curve that most closely relates to the bending strength of the specimen. Second, this relationship does not seem to be sensitive to the data set used, i.e. Integrated Puncture Score accurately captures the correct features for both a wide array of hybrids as well for elite hybrids.

### Integrated Puncture Score can differentiate the strength of hybrids

**Fig. 5:**
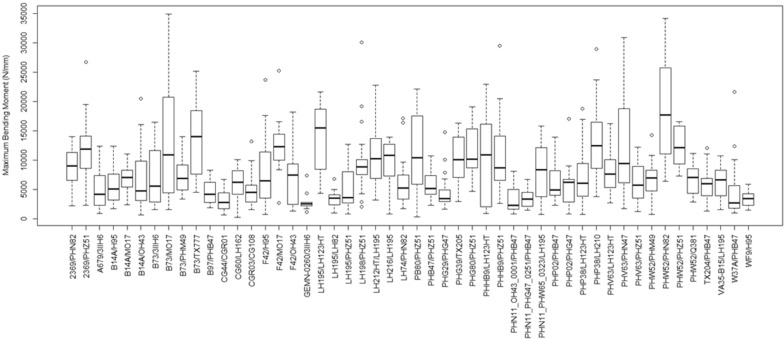
Boxplots of stalk bending strength of the Diversity Set, by hybrid

**Fig. 6:**
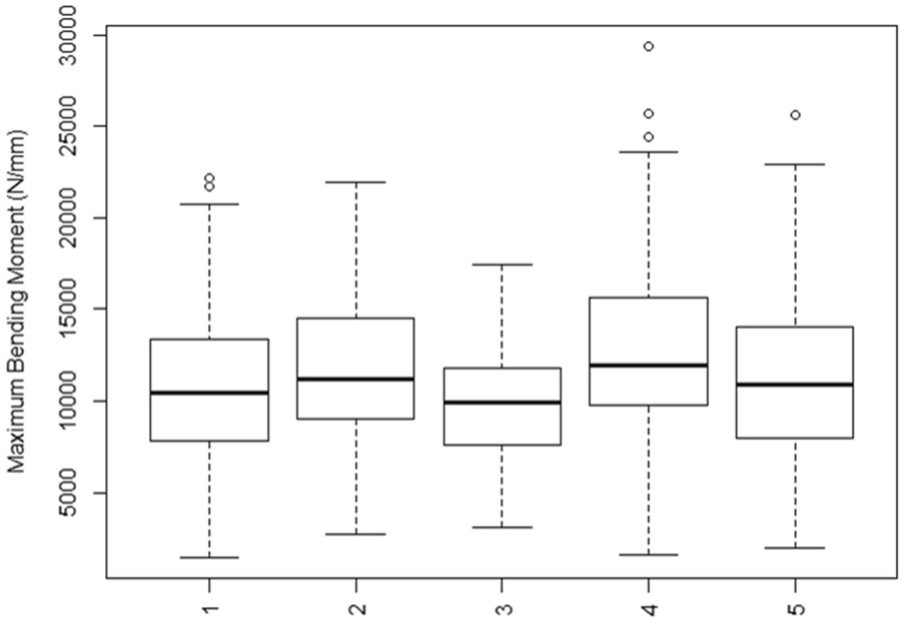
Boxplots of stalk bending strength of the Commercial Set, by hybrid

To test the hypothesis that the Integrated Puncture Score can differentiate the bending strength of hybrids, a series of statistical analyses were performed on the data. Figures 5 and 6 provide a depiction of the variation (via boxplots) in bending strength by hybrid type. As expected, these figures indicate substantial variation in bending strength across hybrids for the Diversity Set, and minimal variation in bending strength across hybrids for the Commercial Set.

Tables 1, 2, 3 and 4 summarize the findings of an ANOVA analysis. In particular, these tables display the ANOVA results as obtained from the *anova* function in R; which present the usual sequential sums of squares, where p-values are for the tests that compare the models against one another in the order specified. From these results we find that hybrid type and plot are highly significant for log-strength for the Diversity Set. It should be noted that the plot variable describes the specific mesocosm, including location of planting, location within the field, and planting density. These findings indicate that there are significant genetic (i.e., hybrid type) and mesoscale (i.e. plot) effects that are still not captured with either Integrated Puncture Score or rind puncture resistance. Standard model diagnostics (e.g., residual plots, QQ-plots, etc.) were conducted to assess the validity of each of these models.

**Table 1:** ANOVA analysis of Integrated Puncture Score, hybrid, and plot predicting bending strength, Diversity Set

	Df	Sum Sq	Mean Sq	F-statistic	*P* value
Integrated Puncture Score	1	353.9	353.9	2954	< 2.2e−16
Hybrid	49	62.79	1.280	10.70	< 2.2e−16
Plot	48	24.26	0.5100	4.220	< 2.2e−16
Residual	742	88.87	0.1200

**Table 2:** ANOVA analysis of rind puncture resistance, hybrid, and plot predicting bending strength, Diversity Set

	Df	Sum Sq	Mean Sq	F-statistic	*P* value
Rind puncture resistance	1	355.4	355.4	2419	< 2.2e−16
Hybrid	49	40.93	0.8400	5.683	< 2.2e−16
Plot	48	24.38	0.5100	3.457	4.38E−13
Residual	742	109.0	0.1500

**Table 3:** ANOVA analysis of Integrated Puncture Score, hybrid, and plot predicting bending strength, Commercial Set

	Df	Sum Sq	Mean Sq	F-statistic	*P* value
Integrated Puncture Score	1	123.0	123.0	3682	< 2.2e−16
Hybrid	4	2.998	0.7500	22.45	< 2.2e−16
Plot	92	12.26	0.1330	3.991	< 2.2e−16
Residual	835	27.88	0.03300

**Table 4:** ANOVA analysis of rind puncture resistance, hybrid, and plot predicting bending strength, Commercial Set

	Df	Sum Sq	Mean Sq	F-statistic	*P* value
Integrated Puncture Score	1	80.01	80.01	1272	< 2.2e−16
Hybrid	4	2.937	0.7340	11.67	3.16E−09
Plot	92	30.64	0.3330	5.294	< 2.2e−16
Residual	835	52.53	0.06300		

## Discussion

Results demonstrate the Integrated Puncture Score methodology provides several advantages as compared to the traditional rind penetration technique. In particular, as hypothesized, the Integrated Puncture Score is better able to distinguish the bending strength of elite hybrids as compared to the traditional method. This is because the Integrated Puncture Score accounts for key determinants of stalk bending strength that traditional puncture methods do not account for. For example, stalk bending strength is ultimately determined by 2 key characteristics: (1) the material properties of the stalk tissues and (2) the geometry of the stalk (e.g., the stalks section modulus, diameter, rind thickness etc.[33, 36]). While both geometry and material properties are important prior research has shown that the geometry of the stalk is more influential on bending strength as compared to material properties [32, 33, 36]. Interestingly, the traditional rind penetration method measures puncture force (i.e., a material property of the rind tissue) but does not account for key geometric features of the stalk (e.g., diameter) which are more influential. In fact, prior research indicates that using traditional rind penetration tests as a breeding metric produces stalks with smaller diameters [15]. Thus, using traditional puncture tests as a breeding metric may produce plants with stronger stalk material properties but weaker stalk geometries. The Integrated Puncture Score on the other hand accounts for both the material properties of the stalk (i.e., puncture force of rind and pith materials) as well as the cross-sectional distribution of the stalk’s structural materials (i.e., geometry). This allows for the calculation of a number that accounts for both the heterogeneity of the material and the morphology of the stem, both of which contribute to the lodging resistance of the stalk.

While the Integrated Puncture Score is a better predictor of stalk bending strength than traditional rind penetration tests the method does have some drawbacks. For example, the Integrated Puncture Score is slightly more damaging to the plant as it requires puncturing through the entirety of the stalk cross-section as opposed to just half of the stalk cross-section. Additionally, a larger diameter probe made of high strength steel is required when utilizing the Integrated Puncture Score method to prevent the probe from bending or breaking. The method also requires collection of synchronous load–displacement data. Currently there are no field based phenotyping devices capable of collecting synchronous load–displacement data from puncture tests. The authors are currently working to develop such a device to enable Integrated Puncture Score measurements to be taken on live plants in the field. This would prevent the need to transport stalks to a laboratory for testing as was done in this study.

Several alternative approaches of analyzing the synchronous load–displacement data from a stalk puncture test were investigated as a part of this study. These included metrics such as the slope and size of different regions of the load–displacement curve, the area under different regions of the curve, as well as several data transformations and adaptations of the Integrated Puncture Score equation. Most of these metrics and transformations were partially informed by engineering theory. However, from a structural engineering standpoint the most appropriate way in which to relate the load–displacement data from a puncture test to bending strength is by means of the Integrated Puncture Score. Indeed, the predictive ability of the Integrated Puncture Score outperformed any other amalgamation of load–displacement data the authors could construe. Nonetheless to further examine the possibility of an alternative yet superior method of utilizing load–displacement data from a puncture test to predict bending strength an empirical functional regression analysis was conducted. The resulting empirical model was highly correlated (R^2^ > 0.90) with the Integrated Puncture Score. These results suggest that neither empirical nor phenomenological relationships are more associated with the bending strength of stalks than pure engineering theory (i.e., the Integrated Puncture Score). This in turn suggests that future research should focus on improving the physical setup of puncture tests and on minimizing sources of measurement error as opposed to attempting to improve the analysis and/or post processing of puncture test data.

Several improvements may yet be realized with respect to the experimental setup of stalk puncture tests. For example, in this study a chamfered probe geometry was employed as it was shown to work well in previous studies [14, 29]. However, it remains to be determined if an alternative probe geometry may provide a better relationship with stalk bending strength. In addition, the puncture rate (i.e., speed of the penetrating probe) was held constant in current study. While it is commonly accepted that the puncture rate affects test results no detailed studies have been conducted to determine what puncture rate may be most appropriate. Future studies should be careful to publish the probe geometry and puncture speed utilized in the study. In addition, parametric analyses which simultaneously vary both puncture rate and probe geometry are needed. Because the puncture rate was held constant in the current study, it remains unclear if the Integrated Puncture Score works best by integrating the load–displacement (work) or the time-displacement (energy) data curve. In summary, the experimental setup of puncture tests should not be overlooked and should continue to be investigated and improved in the future. Previous studies into the biomechanics of stalk lodging have revealed non-intuitive confounding factors that can hamper experimental measurement efforts [37, 38, 42] and similar non-intuitive factors may affect puncture test results.

While the Integrated Puncture Score is strongly related to stalk bending strength, it should be noted that any puncture test is simply unable to simultaneously account for all determinants of stalk bending strength. For example, the Integrated Puncture Score accounts for cross-sectional distribution of structural material within the stalk but it does not account for how the material may be distributed longitudinally along the length of the stalk. Previous studies have demonstrated the importance of longitudinal tissue distribution (i.e., stalk taper) and that many genotypes exhibit structurally inefficient tapers [34]. Additionally, other studies have indicated geometric features known as stress concentrators can significantly affect stalk bending strength [36]. Neither geometric stress concentrators nor the efficiency of the stalk taper is accounted for by a single puncture test. Other factors that influence stalk lodging resistance include stem strength, stem wall thickness, plant height, ear height, flexural stiffness, and the gradient distribution of internal fiber bundles [13]. The authors expect that the Integrated Puncture Score is correlated with some of these factors but certainly not all of them (e.g., plant height and ear height). Also of note is that puncture tests do not induce natural loading patterns on plants and therefore do not produce natural stalk lodging failure patterns in large grain crops [43]. For example, when maize plants stalk lodge they exhibit a distinct creasing failure that occurs just above the node [43, 44]. The most accurate devices for phenotyping stalk lodging resistance should ideally induce natural loads and failure patterns. Additionally, results from this study indicated that genotype and environment significantly related to the bending strength of stalks even after accounting for the Integrated Puncture Score.

### Limitations

Inherent to Integrated Puncture Score formulation is the assumption that the maize stem is circular and symmetrical about its midpoint (Fig. 1c, Point D), with a diameter equal to the measured minor diameter of the stalk. Although maize stems are elliptical, previous work has shown that the major and minor diameters are highly correlated [20]. As such, the major diameter can be reasonably approximated by the minor diameter multiplied by a constant. Substituting this into the elliptical section modulus equation causes it to reduce to the equation for the section modulus of a circle multiplied by a constant. However, constants have no effect on linear regression analyses. We therefore chose not to include the constant term in our analyses and simply utilized the equation for the section modulus of a circle when formulating Eq. 2.

All puncture test methodologies used for assessing lodging resistance are based on the assumption that the plant’s fracture mechanics in the transverse direction are somehow related to the tissue properties of the plant in the longitudinal direction [38]. However, a full mechanistic investigation into the exact relationship between the transverse fracture mechanics and the longitudinal elastic tissue properties of plants is required to more deeply understand the governing physics of this phenotyping approach. Such an investigation would allow researchers to better understand how parameters like probe geometry, probe speed, stem morphology, tissue type, plant type etc. affect puncture test results. This would enable researchers to optimize these parameters for their specific application or study.

In the current study the Integrated Puncture Score was utilized to predict stalk bending strength. However, other scientists have shown that puncture tests may also be a viable manner in which to phenotype for pest and disease resistance [16, 45]. Future studies should investigate the relationship between pest and disease damage (e.g., stalk rot diseases) and features of the load–displacement data curve produced during puncture tests of maize stalks.

Finally, this study was performed on dried stalk specimens. As such results from this study are applicable to late season stalk lodging but may not be applicable to early season lodging (also known as green snap) or root lodging. Further testing is required to determine if the Integrated Puncture Score method can be used successfully on green specimens, and how various factors (e.g. time-of-day, turgor pressure, biotic and abiotic stressors) influence the relationship between Integrated Puncture Score and stalk bending strength and lodging resistance.

## Conclusions

The ability for plant breeders and agronomists to perform high-throughput phenotyping of stalk strength and stalk lodging resistance is still lacking. The first step in developing such a phenotyping program is to develop the testing protocol. The Integrated Puncture Score presented in this study is strongly associated with stalk bending strength and is therefore a good candidate for future high-throughput phenotyping studies. To the best of the authors’ knowledge, this is the first study to examine the entire rind penetration load–displacement curve, using the richness of the dataset to produce a physics-informed numerical score for stalk bending strength. Additionally, the strong agreement between the Integrated Puncture Score and the empirical model supports the claim that the presented method provides reasonable results. The Integrated Puncture Score can also differentiate between elite hybrids, potentially providing plant breeders with tools for phenotypic differentiation late in the breeding process.

## Supplementary information

**Additional file 1.** Matlab algorithm used to calculate the integrated puncture score.

**Additional file 2.** Example force-displacement data from a puncture test.

**Additional file 3.** Example force-displacement data from a puncture test.

**Additional file 4.** Example force-displacement data from a puncture test.

**Additional file 5.** Example force-displacement data from a puncture test.

**Additional file 6.** Example force-displacement data from a puncture test.

## Data Availability

The datasets used and/or analyzed during the current study are available from the corresponding author on reasonable request.
